# Relationship between serum ferritin and pro-inflammatory markers in late pregnancy: An exploratory analysis from Cartagena, Colombia

**DOI:** 10.7705/biomedica.7467

**Published:** 2025-03-28

**Authors:** Alejandra Puerto, Nelson Rafael Alvis-Zakzuk, Walter Annicchiarico, Nelson Alvis-Guzmán, Josefina Zakzuk

**Affiliations:** 1 Instituto de Investigaciones Inmunológicas, Universidad de Cartagena, Cartagena de Indias, Colombia Universidad de Cartagena Universidad de Cartagena Cartagena de Indias Colombia; 2 Línea de Salud Materna, ALZAK Foundation, Cartagena de Indias, Colombia ALZAK Foundation Cartagena de Indias Colombia; 3 Medicina Materno-Fetal, Universidad Pontificia Bolivariana, Bogotá, D. C., Colombia Universidad Pontificia Bolivariana Universidad Pontificia Bolivariana Bogotá D. C. Colombia; 4 Ciencias de la Salud, Universidad de la Costa, Barranquilla, Colombia Universidad de la Costa Barranquilla Colombia

**Keywords:** Anemia, infant, very low birth weight, obstetric labor, premature, pregnancy, inflammation, cytokines, interleukin-6, interleukin-8., anemia, recién nacido de muy bajo peso, trabajo de parto prematuro, embarazo, inflamación, citocinas, interleucina-6, interleucina-8

## Abstract

**Introduction.:**

In a previous study, we identified an inverse relationship between adverse perinatal outcomes and iron status during late pregnancy of women recruited from a maternal hospital in Cartagena, Colombia. Some of these outcomes have also been linked to maternal inflammatory states. However, there is currently no clarity regarding the relationship between iron levels and proinflammatory markers during this period.

**Objective.:**

To estimate the relationship between inflammatory markers and serum ferritin in third-trimester pregnancies.

**Materials and methods.:**

Serum ferritin, hemoglobin, and proinflammatory cytokine levels were determined in women in Cartagena in their third trimester of pregnancy. We analyzed the relationship between ferritin levels and proinflammatory cytokines, as well as the relationship between serum ferritin, hemoglobin, and inflammatory cytokine levels with adverse perinatal outcomes.

**Results.:**

The levels of IL-6 were significantly associated with serum ferritin levels (β = 0.42, SE = 0.21, p = 0.04) but not with maternal age. Maternal serum ferritin had a positive weak correlation with the absolute number of lymphocytes and monocytes. Hemoglobin and maternal serum ferritin were weakly and inversely associated with birth weight. Serum ferritin but not IL-6 or IL-8 was associated with preterm birth.

**Conclusions.:**

We observed direct and mild associations of serum iron markers (serum ferritin, hemoglobin, and hematocrit) with lymphocyte counts. The inflammation marker, IL- 6, was mildly associated with serum ferritin levels in late pregnancy. Women with elevated white blood cell counts and serum ferritin levels tended to have infants with lower birth weights. This fact suggests a potential involvement of iron in inflammatory processes during pregnancy, and conditions associated with inflammation in the final trimester may have adverse effects on perinatal outcomes.

## Resumen

**Introducción.:**

En un estudio previo, se identificó una relación inversa entre los resultados perinatales adversos y el estado del hierro -al final del embarazo- en mujeres reclutadas en un hospital materno de Cartagena, Colombia. Algunos resultados también se han vinculado a estados inflamatorios maternos. Sin embargo, no hay claridad respecto a la relación entre los niveles de hierro y los marcadores proinflamatorios durante este período.

**Objetivo.:**

Estimar la relación entre los marcadores inflamatorios y la ferritina sérica en el tercer trimestre de gestación.

**Materiales y métodos.:**

Se determinaron los niveles de ferritina sérica, hemoglobina y citocinas proinflamatorias en mujeres gestantes de Cartagena. Se analizó la relación entre la ferritina sérica y las citocinas proinflamatorias, así como la relación entre los marcadores de hierro e inflamación y los resultados perinatales adversos.

**Resultados.:**

Los niveles de IL-6 tuvieron una asociación estadísticamente significativa con la concentración de ferritina sérica (β = 0,42, DE = 0,21, p = 0,04). Hubo una correlación positiva débil entre la ferritina sérica materna y el recuento de linfocitos y monocitos.

**Conclusiones.:**

Los marcadores de hierro en sangre (ferritina sérica, hemoglobina y hematocrito) están directamente relacionados con el número de linfocitos. El marcador de inflamación, IL-6, está asociado con los niveles de ferritina sérica en el último trimestre del embarazo. Las mujeres con mayor número de leucocitos y niveles elevados de ferritina sérica tuvieron hijos con menor peso al nacer. Esto sugiere una posible participación del hierro en los procesos inflamatorios durante el embarazo y que las condiciones asociadas con la inflamación en el último trimestre podrían tener efectos adversos en los resultados perinatales.

Iron is an essential element for fetal growth. Therefore, markers of iron status (such as hemoglobin and ferritin) are monitored during prenatal care ^1^. In low- and middle-income countries, where food insecurity and gestational anemia are more prevalent, universal iron supplementation is included in health services during pregnancy ^2^. Public health strategies have primarily focused on assessing and addressing iron deficiency, given the clear association between iron deficiency -especially in the first trimester- and adverse outcomes such as low birth weight and preterm birth ^3^. However, it is important to mention that higher levels of hemoglobin and serum ferritin have been related to a higher risk of adverse pregnancy outcomes ^4^^-^^8^. To date, scientific evidence supports a U curve pattern in the relationship between iron status and the occurrence of perinatal and maternal clinical adverse outcomes, where extremes can be detrimental to the health of the mother and the fetus ^9^^-^^16^.

In a previous study, we found an inverse relationship between birth weight and gestational age with maternal serum ferritin in the third trimester of pregnancy in women recruited at a maternity hospital in Cartagena, Colombia ^17^. Like our study, research in countries such as Ghana ^18^^,^^19^, China, Papua New Guinea ^20^, and South Africa show the same trend for iron effects.

The involvement of iron in inducing proinflammatory states has been documented. Reports suggest that elevated supplemental iron levels during pregnancy, or even physiological values, could negatively affect maternal health ^21^^-^^23^. Nevertheless, studies focused on the relationship between maternal iron status and inflammation are scarce ^24^^,^^25^. Different studies correlate gestational anemia and serum ferritin deficiency with outcomes such as low birth weight, preterm birth, and alterations in fetal growth, as well as the relationship between higher iron levels and inflammatory states. However, no clarity exists regarding the relationship between iron levels and proinflammatory markers during pregnancy and its potential association with adverse maternal and perinatal outcomes.

Changes in the immune system during pregnancy are primarily associated with augmented type 2 cellular immunity aiming to regulate proinflammatory activity. This physiological adaptation leads to increased production of regulatory interleukins, such as interleukin-10 (IL-10), resulting in a decrease in proinflammatory activity (e.g. IL-6). Consequently, this reduction helps to mitigate the risk of rejection of the implanted trophoblast ^26^^-^^28^. Hence, an increase in the activity of proinflammatory interleukins during pregnancy can be considered a pathological scenario associated with unwanted maternal and perinatal outcomes, including alterations in fetal growth and preeclampsia, among others ^29^^-^^31^.

Therefore, we aimed to evaluate the relationship between white blood cells, serum proinflammatory cytokines (IL-6, IL-8, IL-12p), and serum ferritin levels in a pregnant population during their third gestational trimester. This knowledge may impact on the prevention and treatment of inflammatory conditions during pregnancy by helping to clarify whether there are direct relationships between elevated levels of this nutrient and systemic

inflammation. It also encourages further research into appropriate iron supplementation during pregnancy and its widespread administration to prevent low birth weight in developing countries.

## Materials and methods

### Study design and population

This study involves a subsample of 530 participants selected from pregnant patients enrolled in a birth cohort study to analyze the relationship between white blood cell counts and iron markers ^17^. Participants were recruited from the *E.S.E. Clínica Maternidad Rafael Calvo C*., a referral hospital in Cartagena, Colombia, which provides obstetric care for the entire department of Bolívar.

Following an extreme group approach design, cytokine analyses were performed on a subset of these participants (n = 117), classified based on their serum ferritin concentration -low (percentile < 25) and high (percentile > 75)-, obtained from a serum bank established for this cohort. The research was approved by the ethics committee of the clinic (authorization number: 001-18).

### Eligibility criteria and enrollment process

The ancillary study included pregnant women residing in the department of Bolivar, aged between 18 and 45 years, in the last trimester of pregnancy and in the first stage of labor at the time of recruitment. For this secondary analysis, the included participants were required to have complete hemogram and leukogram data available, a normal pregnancy, and no signs of active infections.

Exclusion criteria included mothers diagnosed with HIV, autoimmune diseases, diabetes mellitus, chronic kidney disease, or previous history of hypertensive disorder during pregnancy. Additionally, mothers of newborns with congenital defects, TORCH syndrome, or chromosomal abnormalities were excluded from the study.

### Blood samples

Blood was collected from patients during the recruitment phase. Hemogram and serum ferritin determinations were performed as previously described ^17^. Serum was isolated and stored at -80°C for future use. Serum levels of IL-Ιβ, IL-6, IL-8, IL-10, TNF-α, and IL-12 were measured by flow cytometry using the Human Inflammatory Cytokine Cytometric Bead Array™ kit, following the manufacturer’s instructions (BD, New Jersey, USA).

### Data analysis

Frequency rates and inferential analyses were estimated using Statistical Package for Social Sciences software (SPSS™, version 25.0; IBM). Since most variables were not normally distributed, they were reported as the median and interquartile range. Leukogram, iron status markers, and inflammatory cytokine data were log-transformed and then analyzed using parametric tests. White blood cell populations were assessed in relation to iron markers (hemoglobin, hematocrit, red blood cells, and serum ferritin) and neonatal anthropometric data using Pearson correlation tests. Correlograms were generated using the “corrplot” package in R Studio™ (version 4.3). Significant correlations were further evaluated in linear regression models and included maternal age, socioeconomic status, and gestational age. Iron markers served as covariates. A p value inferior to 0.05 was considered significant for all tests.

For cytokine measurements, we selected 117 women at the extremes of the distribution (≤ 25^th^ percentile or “low” and ≥ 75^th^ percentile or “high”) of serum ferritin values. Cytokine levels were log-transformed and compared between the two groups using a t test for independent groups. A p value inferior to 0.05 was considered significant for all tests.

## Results

Sociodemographic characteristics and health-related conditions during pregnancy for the 530 women included in this study are shown in table 1. The median serum ferritin value was 15.2 μg/L (IQR = 8.7 - 25.05). Only one participant presented hyperferritinemia (serum ferritin > 200 pg/L) ^32^.

**Table 1:** Sociodemographic characteristics and health-related conditions during pregnancy of the sample study (N = 530)

Characteristics		
Age (mean ± SD)		24.6 ± 5.3
Living in an urban area [n (%)]		428 (80.8)
Socioeconomic status		[n (%)]
	1	494 (93.2)
	2	28 (5.3)
	3	7 (1.3)
Blood count		(mean ± SD)
	Hemoglobin (g/dl)	11.0 ± 1.3
	Hematocrit	33.9 ± 3.7
	White blood cells	11,507.9 ± 3,429.4
	Granulocytes (%)	72.6 ± 10.0
	Monocytes (%)	5.2 ± 2.0
	Lymphocytes (%)	20.2 ± 7.5
Iron status		[n (%)]
	Gestational anemia	229 (43.2)
	Serum ferritin depletion	213 (40.2)
Delivery mode		
	Cesarean section	243 (45.8)
Newborn		
	Gestational age at birth (mean ± SD)	38.5 ± 1.9
	Birth weight (mean ± SD)	3,134.9 ± 494.4
	Birth size (mean ± SD)	50.3 ± 3.1

**Figure 1:**
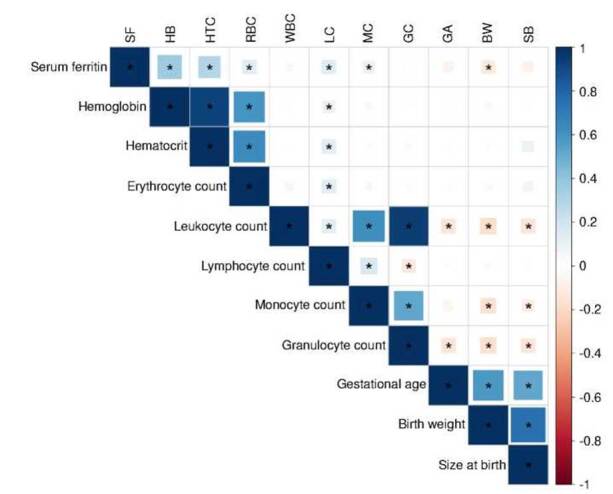
Correlation of serum ferritin, blood cell populations, and maternal-perinatal variables of interest. The scale represents Pearson's coefficient (R), ranging from -1 to +1. Positive correlations are indicated using cool colors on the scale.

SF: Serum ferritin; HB: Hemoglobin; HTC: Hematocrit; RBC: Red blood cells; WBC: White blood cells; LC: Lymphocyte count; MC: Monocyte count; GC: Granulocyte count; GA: Gestational age; BW: Birth weight; SB: Size at birth

Under the hypothesis that high iron levels may promote inflammatory states, we explored the relationship between various immune markers and indicators of iron status in the participants. As observed in figure 1, we found significant and positive weak correlations between the absolute lymphocyte count and hemoglobin (r = 0.09, p = 0.02), hematocrit (r = 0.13, p = 0.003), red blood cells (r = 0.13, p = 0.004), and serum ferritin (r = 0.12, p < 0.001). Monocytes also correlated with serum ferritin (r = 0.09, p < 0.001).

* Significant correlations (p < 0.05)

In separate multivariate linear regression models, we observed that lymphocyte and monocyte counts were associated with serum ferritin, considering maternal age, socioeconomic status, and gestational weeks as covariates (table 2). Interestingly, white blood cells inversely correlated with birth weight (r = -0.17, p < 0.001). When this relationship was evaluated by linear regression, the variables remained associated even after adjustment for maternal age, gestational age, and socioeconomic status.

**Table 2:** Relationship between serum ferritin levels with leukocyte counts in pregnant women

Variable		β	SE	p value
Lymphocyte counts				
	Maternal age	0.031	0.069	0.478
	Socioeconomical status	-0.049	0.041	0.263
	Gestational age	-0.010	0.238	0.813
	Serum ferritin	0.123	0.017	0.005
Monocyte counts				
	Maternal age	-0.189	0.097	<0.0001
	Socioeconomic status	0.025	0.058	0.565
	Gestational age	-0.054	0.337	0.206
	Serum ferritin	0.092	0.024	0.032

β: Beta coefficient; SE: Standard error

Predictors associated with the dependent variable (p < 0.05) are highlighted in bold.

### Maternal serum ferritin and IL-6 levels

**Figure 2:**
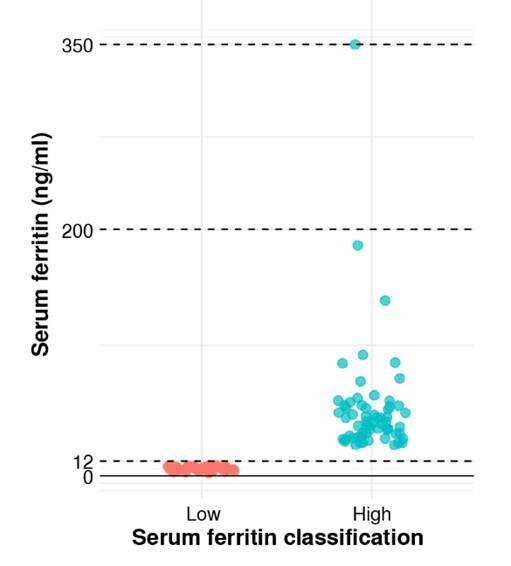
Serum ferritin concentrations. Subjects were classified into two groups based on ferritin levels: low ferritin and high ferritin.

**Figure 3:**
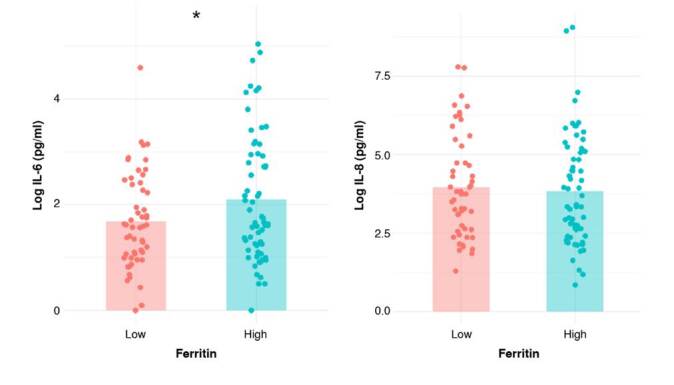
Cytokine levels by serum ferritin group. Graphs display mean levels of interleukin-6 (IL- 6) and interleukin-8 (IL-8) in participants categorized into low and high serum ferritin groups. Data are presented as the means of logarithmically transformed values.

We used an extreme group approach design and compared cytokine levels between women with low and high serum ferritin. The distribution of serum ferritin levels in both groups is shown in figure 2, and the sociodemographic characteristics of the participants are presented in table 3. IL-Ιβ levels were undetectable in all patients, while TNF-α was detected in only one, IL-12p in twelve patients, and IL-10 in two women. Only IL-6 and IL-8 were detected in the serum of most participants. Log-transformed IL-6 values were significantly higher in the “high” serum ferritin group (1.68 ± 0.89 pg/ml versus 2.10 ± 1.21 pg/ml, p = 0.03) (figure 3). IL-6 values were significantly associated with serum ferritin status after adjustment for gestational age, maternal age, and socioeconomic status (β = 0.42, SE = 0.21, p = 0.04). No significant differences in IL-8 levels were observed between groups (3.96 ± 1.64 pg/ml versus 3.84 ± 1.76 pg/ml).

**Table 3:** Participants’ descriptive characteristics by ferritin subgroup, including cytokine data

		Low serum ferritin (Q1)	High serum ferritin (Q4)
		(N = 53)	(N = 63)
Maternal			
	Age (mean ± SD)	23.51 (4.83)	24.70 (5.52)
	Living in an urban area [n (%)]	42 (79.2)	52 (81.3)
Socioeconomic status		[n (%)]	[n (%)]
	1	48 (90.6)	59 (92.2)
	2	4 (7.5)	4 (6.3)
	3	1 (1.9)	1 (1.6)
Blood count		(mean ± SD)	(mean ± SD)
	Hemoglobin (g/dl)		10.4 ± 1.1	11.7 ± 1.5
	Hematocrit		32.1 ± 3.2	35.2 ± 4.8
	White blood cells		10,684.9 ± 3,456.5	11,412.67 ± 3,861.29
	Granulocytes		72.42 ± 10.64	73.07 ± 9.45
	Monocytes			5.21 ± 1.93
	Lymphocytes			19.64 ±6.19
Iron status			
	Gestational anemia [n (%)]		30 (56.6)	17 (26.6)
	Serum ferritin (median ± IQR)		5.90 (5.0 - 7.3)	43.1 (32.1 - 56.9)
Cytokines		(pg/ml)	(pg/ml)
	IL-6 (median ± IQR)		5.85 (4.00 - 13.21)	8.59 (4.06 - 26.24)
	IL-8 (median ± IQR)		41.29 (12.67 - 173.48)	28.99 (12.80 - 171.13)
	IL-12p (median ± IQR)		2.48 (2.32 - 7.30)	2.09 (1.92 - 2.47)

SD: Standard deviation; IQR: Interquartile range; IL-6: Interleukin-6; IL-8: Interleukin-8; IL-12p:

Interleukin-12p

IL-12p levels were detectable in 12 women.

* p < 0.05

No association was found between the concentration of IL-6 or IL-8 with birth weight (β = -0.00, SE = 0.00, p = 0.44), birth length (β = 0.04, SE = 0.03, p = 0.14), or gestational age of the participants’ children (β = -0.04, SE = 0.06, p = 0.48).

## Discussion

Understanding iron balance during pregnancy remains a complex issue. While much nutritional advice during pregnancy focuses on preventing anemia to ensure fetal health ^33^, previous findings, as in other underdeveloped countries, have indicated an inverse association between serum ferritin and hemoglobin levels with adverse fetal outcomes such as low birth weight ^11^^,^^18^. Given the hypothesis that iron may contribute to inflammatory states, we investigated, for the first time in the Colombian population, the relationship between serum ferritin and various inflammatory markers, including white blood cell counts and serum proinflammatory cytokine levels.

The relationship between iron status, inflammatory markers, and pregnancy outcomes has been primarily investigated in preeclampsia, where higher neutrophil and lymphocyte counts and elevated serum iron and ferritin concentrations have been detected in pregnant women with this condition compared to those with healthy pregnancies ^34^^,^^35^. This phenomenon may be attributed to endothelial dysfunction from oxidative stress secondary to increased maternal iron levels.

Although Lee *et al*. used a different study design than ours, they found that IL-6 concentrations at delivery were positively correlated with maternal serum ferritin (p < 0.01) ^24^. However, the relationship between maternal serum ferritin and IL-6 is controversial, as other studies have found no association between them in healthy or high-risk pregnancies, which often include autoimmune diseases, obesity, diabetes mellitus, and preeclampsia ^25^^,^^36^. Nevertheless, studies evaluating the correlation between serum ferritin and interleukins in pregnant patients have small sample sizes, limiting the extrapolation of the results.

Similar findings have been published regarding white blood cells and iron status. Tang *et al*. found a direct association between hemoglobin concentration and T-cell count in healthy third-trimester pregnant women ^37^. Consistent with the aforementioned findings, some studies reported non pregnant individuals with anemia exhibiting lower counts of lymphocytes and natural killer cells ^38^. However, the precise mechanism of how hemoglobin levels influence peripheral lymphocytes is not completely understood. Some white blood cell populations demonstrate functional alterations in anemic states, such as diminished bactericidal activity in macrophages ^39^ or reduced enzymatic activity in neutrophils (in mice) ^40^.

Iron participates in the immune response by inducing immune cell differentiation and regulating cellular response pathways and cytokine functions ^41^. High white blood cell counts may result from augmented cell proliferation facilitated by iron availability ^42^. Moreover, under such conditions, macrophage M1 polarization is induced by inflammatory cytokines such as IL-6 or TNF-α, along with increased expression of other iron metabolism proteins, such as ferroportin, transferrin receptor, and ferritin ^43^^,^^44^. Similarly, high iron levels may enhance neutrophil recruitment and promote inflammatory states through the secretion of proinflammatory cytokines, as evidenced in patients with hemochromatosis ^45^.

White blood cell values represent the activity of the immune response and have been identified as predictive factors for some chronic inflammatory conditions ^46^^-^^49^. Although white blood cell values increase physiologically during late pregnancy ^50^, its count may be used in predicting adverse perinatal events, such as preterm birth and preeclampsia severity ^51^^-^^54^. Consistent with this, the white blood cell count was inversely related to birth weight and gestational age. This result complements previous findings in this cohort, where serum ferritin was inversely associated with birth weight ^17^. Similar findings have been reported in Israeli pregnant women, in whom the white blood cell count also showed an inverse association with birth weight ^55^ and is related to adverse perinatal outcomes ^56^^-^^58^. The previous information suggests a potential connection between maternal iron levels and inflammatory immune activity, which could have adverse effects on perinatal outcomes.

Our results suggest that maternal iron status before delivery could influence the systemic inflammatory response. Studies on the proinflammatory effects of serum ferritin have identified that stimulation of macrophages with the heavy subunit (FeH) of ferritin induced a significant increase in the mRNA expression of IL-Ιβ, IL-6, IL-12, and TNF-α, as functionally verified -by ELISA- with elevated levels of IL-1 β and IL- 12p70 secreted by macrophages ^59^. These results could suggest a proinflammatory role of elevated iron levels during pregnancy, negatively impacting maternal-fetal outcomes.

IL-6 -secreted by monocytes, macrophages, and T cells in response to recognition of pathogen-associated molecular patterns- stimulates C-reactive protein and hepcidin hormone secretion by hepatocytes through the involvement of the transcription factor STAT3 and NF-ke ^60^^,^^61^. C-reactive protein, in turn, induces the release of reactive oxygen species and cytokines by macrophages ^62^. Inflammatory states within placental tissue may trigger the production of IL-6 by circulating monocytes or macrophages, initiating a systemic-type response. This response includes the release of cytokines, such as IL-1, TNF-α, IFN-γ, or IL-6 in the serum. Consequently, these changes result in decreased serum iron levels (hypoferremia) ^63^, leading to iron sequestration intracellularly or in circulating storage proteins, such as ferritin, or increased storage in macrophages of the reticuloendothelial system ^64^.

Despite the results of our study, it is important to highlight that outcome such as low birth weight are usually not acute. Therefore, the findings of this study could represent the consequences of changes that occurred in the early stages of pregnancy or before ^3^^,^^12^^,^^15^^,^^65^^,^^66^.

Our study has several limitations. Enrollment of participants in the peripartum implies higher cytokine levels observed due to their physiological increase during this period. Thus, the measurements do not represent the third trimester of pregnancy ^67^^,^^68^. Nevertheless, we excluded participants from the ancillary cohort with comorbidities associated with systemic inflammation. Other causes, such as obesity, were not analyzed in this study, and may have resulted in a biased interpretation of the results.

We also recognize the limitations of the extreme group approach design in determining effect size, although it is informative for identifying a positive association between iron markers and proinflammatory conditions in late pregnancy ^69^.

Additionally, the study design does not allow for causality, as we cannot ascertain whether iron overload is a stimulus to produce proinflammatory cytokines or, on the contrary, if inflammation induces the elevation of serum ferritin during pregnancy. The multiplex cytokine assay used was not sensitive enough to detect blood levels of IL-10, an anti-inflammatory cytokine relevant in fetal tolerance, associated with iron-deficiency anemia in the adult population ^70^.

Further studies are required to functionally corroborate the influence of iron overload on maternal serum and tissue (placenta) inflammation. In addition, serum ferritin can be affected by the expansion of plasma volume, so other indicators of iron status, such as the concentration of the soluble transferrin receptor, could better represent the maternal iron status.

In summary, in a representative sample of pregnant women in Cartagena, Colombia, iron markers (serum ferritin, hemoglobin, and hematocrit) are correlated to lymphocyte count and associated with inflammation markers such as IL-6. Furthermore, we observed that women with elevated white blood cell counts and high serum ferritin levels tended to have infants with lower birth weights. This fact suggests a potential involvement of iron in inflammatory processes during pregnancy; conditions associated with inflammation in the final trimester may have adverse effects on perinatal outcomes.

However, further studies are required to establish causality between these events. Also, associations were generally of weak magnitude. Then, other aspects that may have an influential role in systemic inflammation must be identified. The direct effects of iron on the fetoplacental unit should be evaluated in tissue (placenta) for a closer approximation to the biological background of this finding.

## References

[1] World Health Organization WHO guideline on use of ferritin concentrations to assess iron status in individuals and populations.

[2] World Health Organization Guideline: Daily iron and folic acid supplementation in pregnant women.

[3] Rahman MM, Abe SK, Rahman MS, Kanda M, Narita S, Bilano V (2016). Maternal anemia and risk of adverse birth and health outcomes in low-and middle-income countries: Systematic review and meta-analysis, 2. Am J Clin Nutr.

[4] Tamura T, Goldenberg RL, Johnston KE, Cliver SP, Hickey CA (1996). Serum ferritin: A predictor of early spontaneous preterm delivery. Obstet Gynecol.

[5] Goldenberg RL, Tamura T, DuBard M, Johnston KE, Copper RL, Neggers Y (1996). Plasma ferritin and pregnancy outcome. Am J Obstet Gynecol.

[6] Lao TT (2000). Third trimester iron status and pregnancy outcome in non-anaemic women: Pregnancy unfavourably affected by maternal iron excess. Hum Reprod.

[7] Chen X, Scholl TO, Stein TP (2006). Association of elevated serum ferritin levels and the risk of gestational diabetes mellitus in pregnant women: The Camden study. Diabetes Care.

[8] Scholl TO (1998). High third-trimester ferritin concentration: Associations with very preterm delivery, infection, and maternal nutritional status. Obstet Gynecol.

[9] Randall DA, Patterson JA, Gallimore F, Morris JM, McGee TM, Ford JB (2019). The association between haemoglobin levels in the first 20 weeks of pregnancy and pregnancy outcomes. PloS ONE.

[10] Dewey KG, Oaks BM (2017). U-shaped curve for risk associated with maternal hemoglobin, iron status, or iron supplementation. Am J Clin Nutr.

[11] Gonzales GF, Steenland K, Tapia V (2009). Maternal hemoglobin level and fetal outcome at low and high altitudes. Am J Physiol Regul Integr Comp Physiol.

[12] Malhotra M, Sharma JB, Batra S, Sharma S, Murthy NS, Arora R (2002). Maternal and perinatal outcome in varying degrees of anemia. Int J Gynecol Obstet.

[13] Shobeiri F, Begum K, Nazari M (2006). A prospective study of maternal hemoglobin status of Indian women during pregnancy and pregnancy outcome. Nutr Res.

[14] Peng Z, Si S, Cheng H, Zhou H, Chi P, Mo M (2022). The associations of maternal hemoglobin concentration in different time points and its changes during pregnancy with birth weight outcomes. Nutrients.

[15] Liu D, Li S, Zhang B, Kang Y, Cheng Y, Zeng L (2022). Maternal hemoglobin concentrations and birth weight, low birth weight (LBW), and small for gestational age (SGA): Findings from a prospective study in northwest China. Nutrients.

[16] Young MF, Oaks BM, Rogers HP, Tandon S, Martorell R, Dewey KG (2023). Maternal low and high hemoglobin concentrations and associations with adverse maternal and infant health outcomes: An updated global systematic review and meta-analysis. BMC Pregnancy Childbirth.

[17] Puerto A, Trojan A, Alvis-Zakzuk NR, López-Saleme R, Edna-Estrada F, Álvarez A (2021). Iron status in late pregnancy is inversely associated with birth weight in Colombia. Public Health Nutr.

[18] Oaks BM, Jorgensen JM, Baldiviez LM, Adu-Afarwuah S, Maleta K, Okronipa H (2019). Prenatal iron deficiency and replete iron status are associated with adverse birth outcomes, but associations differ in Ghana and Malawi. J Nutr.

[19] Chen GD, Li PS, Zhou ZX, Wang HY, Gou XY, Ye SX (2024). Associations of maternal serum concentration of iron-related indicators with birth outcomes in Chinese: A pilot prospective cohort study. Ital J Pediatr.

[20] Fowkes FJI, Moore KA, Opi DH, Simpson JA, Langham F, Stanisic DI (2018). Iron deficiency during pregnancy is associated with a reduced risk of adverse birth outcomes in a malariaendemic area in a longitudinal cohort study. BMC Med.

[21] Vásquez-Molina ME, Corral-Terrazas M, Apezteguia MA, Carmona-Sawasky J, Levario- Carrillo M (2001). Relación entre las reservas de hierro maternas y del recién nacido. Salud Pública Mex.

[22] Mujica-Coopman MF, Brito A, López de Romaña D, Ríos-Castillo I, Coris H, Olivares M (2015). Prevalence of anemia in Latin America and the Caribbean. Food Nutr Bull.

[23] Sosa BEP, Mesa SLR, Correa LMM, López LPM (2009). Indicadores bioquímicos del hierro materno en el tercer trimestre de la gestación y su relación con la antropometría materna y el peso al nacer. Iatreia.

[24] Lee S, Guillet R, Cooper EM, Westerman M, Orlando M, Pressman E (2014). Maternal inflammation at delivery affects assessment of maternal iron status. J Nutr.

[25] Petkova-Marinova T, Ruseva B, Paneva-Barzashka B, Atanasova M, Dragomirova P, Laleva PD (2020). Relationships between hepcidin, interleukin-6 and parameters of iron metabolism in pregnant women. Arch Balk Med Union.

[26] Dutta S, Sengupta P, Liew FF (2024). Cytokine landscapes of pregnancy: Mapping gestational immune phases. Gynecol Obstet Clin Med.

[27] Stokkeland LMT, Giske0degard GF, Stridsklev S, Ryan L, Steinkjer B, Tangeras LH (2019). Serum cytokine patterns in first half of pregnancy. Cytokine.

[28] Cubro H, Kashyap S, Nath MC, Ackerman AW, Garovic VD (2018). The role of interleukin-10 in the pathophysiology of preeclampsia. Curr Hypertens Rep.

[29] Sircar M, Thadhani R, Karumanchi SA (2015). Pathogenesis of preeclampsia. Curr Opin Nephrol Hyperlens.

[30] Spence T, Allsopp PJ, Yeates AJ, Mulhern MS, Strain JJ, McSorley EM (2021). Maternal serum cytokine concentrations in healthy pregnancy and preeclampsia. J Pregnancy.

[31] Kirici P, Çagiran F, Kali Z, Tanriverdi E, Mavral N, Ecin S (2023). Determination of maternal serum pro-inflammatory cytokine changes in intrauterine growth restriction. Eur Rev Med Pharmacol Sci.

[32] Sandnes M, Ulvik RJ, Vorland M, Reikvam H (2021). Hyperferritinemia - A clinical overview. J Clin Med.

[33] World Health Organization WHO recommendations on antenatal care for a positive pregnancy experience.

[34] Mannaerts D, Faes E, Cos P, Briedé JJ, Gyselaers W, Cornette J (2018). Oxidative stress in healthy pregnancy and preeclampsia is linked to chronic inflammation, iron status, and vascularfunction. PLoSONE.

[35] Õlmez F, Oglak S, Behram M, Õzkõse Z, Can E, Ünal Õ (2022). Serum prohepcidin concentrations in preeclamptic pregnant women: An analysis concerning serum iron status markers and compared to healthy pregnant women. East J Med.

[36] Ahmed YIB, Yagoub HS, Hassan MA, Adam I, Hamdan HZ (2023). Maternal serum iron status, hepcidin and interleukin-6 levels in women with preeclampsia. Front Physiol.

[37] Tang YM, Chen XZ, Li GR, Zhou RH, Ning H, Yan H (2006). Effects of iron deficiency anemia on immunity and infectious disease in pregnant women. Wei Sheng Yan Jiu.

[38] AlRajeh L, Zaher A, Alghamdi A, Alsheikh R, AlSultan O (2022). Effects of iron deficiency and its indicators on lymphocyte subsets: A study at King Fahd Hospital of the University, Saudi Arabia. J Blood Med.

[39] Hallquist NA, McNeil LK, Lockwood JF, Sherman AR (1992). Maternal-iron-deficiency effects on peritoneal macrophage and peritoneal natural-killer-cell cytotoxicity in rat pups. Am J Clin Nutr.

[40] Spear AT, Sherman AR (1992). Iron deficiency alters DMBA-induced tumor burden and natural killer cell cytotoxicity in rats. J Nutr.

[41] Nakamura T, Naguro I, Ichijo H (2019). Iron homeostasis and iron-regulated ROS in cell death, senescence, and human diseases. Biochim Biophys Acta Gen Subj.

[42] Ni S, Yuan Y, Kuang Y, Li X (2022). Iron metabolism and immune regulation. Front Immunol.

[43] Sindrilaru A, Peters T, Wieschalka S, Baican C, Baican A, Peter H (2011). An unrestrained proinflammatory M1 macrophage population induced by iron impairs wound healing in humans and mice. J Clin Invest.

[44] DeRosa A, Leftin A (2021). The iron curtain: Macrophages at the interface of systemic and microenvironmental iron metabolism and immune response in cancer. Front Immunol.

[45] Das S, Saqib M, Meng RC, Chittur SV, Guan Z, Wan F (2022). Hemochromatosis drives acute lethal intestinal responses to hyperyersiniabactin-producing Yersinia pseudotuberculosis. Proc Natl Acad Sci USA.

[46] Nakanishi N, Yoshida H, Matsuo Y, Suzuki K, Tatara K (2002). White blood-cell count and the risk of impaired fasting glucose or type II diabetes in middle-aged Japanese men. Diabetologia.

[47] Shin G, Jang K, Kim M, Lee JH, Yoo HJ (2020). Inflammatory markers and plasma fatty acids in predicting WBC level alterations in association with glucose-related markers: A crosssectional study. Front Immunol.

[48] Margolis KL, Manson JE, Greenland P, Rodabough RJ, Bray PF, Safford M (2005). Leukocyte count as a predictor of cardiovascular events and mortality in postmenopausal women: The Women’s Health Initiative Observational Study. Arch Intern Med.

[49] Willems JM, Trompet S, Blauw GJ, Westendorp RGJ, de Craen AJM (2010). White blood cell count and C-reactive protein are independent predictors of mortality in the oldest old. J Gerontol Ser A.

[50] Dockree S, Shine B, Pavord S, Impey L, Vatish M (2021). White blood cells in pregnancy: Reference intervals for before and after delivery. eBioMedicine.

[51] Rewatkar M, Jain S, Jain M, Mohod K (2018). C-reactive protein and white blood cell count as predictors of maternal and neonatal infections in prelabour rupture of membranes between 34 and 41 weeks of gestation. J Obstet Gynaecol.

[52] Ma M, Zhu M, Zhuo B, Li L, Chen H, Xu L (2020). Use of complete blood count for predicting preterm birth in asymptomatic pregnant women: A propensity score-matched analysis. J Clin Lab Anal.

[53] Liao D, Chen L, Li Q, Liu G, Wang W, Li J (2022). Predictive value of the peripheral blood parameters for preeclampsia. Clin Lab.

[54] Liu M, Lin P, Qu M, Zhai R, Zhang L, Zhang L (2022). Neutrophil count is a useful marker to predict the severity of preeclampsia. Clin Exp Hypertens.

[55] Moshe NS, Michaeli J, Shalev L, Ruchlemer R, Farkash R, Samueloff A (2020). Maternal white blood cell count: A guide for the neonatal birth weight estimate at term. Am J Obstet Gynecol.

[56] Srebnik N, Michaeli J, Shalev L, Ruchlemer R, Farkash R, Grisaru-Granovsky S (2021). The maternal leukocyte count at admission for labor is indicative of early maternal postpartum infectious morbidity and adverse neonatal outcome. Eur J Obstet Gynecol Reprod Biol.

[57] Eriç Horasanli J, Alp EC, Bülbül R (2022). Evaluation of complete blood cell count parameters in the diagnosis of threatened preterm labor and premature rupture of membranes. Dubai Med J.

[58] Zeng Y, Li L, Mao M, Liang X, Chen M, Xia Y (2021). Establishment of reference intervals of complete blood count for twin pregnancy. BMC Pregnancy Childbirth.

[59] Ruscitti P, Di Benedetto P, Berardicurti O, Panzera N, Grazia N, Lizzi AR (2020). Pro-inflammatory properties of H-ferritin on human macrophages, ex vivo and in vitro observations. Sci Rep.

[60] Bode JG, Albrecht U, Haussinger D, Heinrich PC, Schaper F (2012). Hepatic acute phase proteins - Regulation by IL-6- and IL-1-type cytokines involving STAT3 and its crosstalk with NF-κβ- dependent signaling. Eur J Cell Biol.

[61] Sobotta S, Raue A, Huang X, Vanlier J, Jünger A, Bohl S (2017). Model based targeting of IL-6-induced inflammatory responses in cultured primary hepatocytes to improve application of the JAK inhibitor Ruxolitinib. Front Physiol.

[62] Zhang Z, Yang Y, Hill MA, Wu J (2012). Does C-reactive protein contribute to atherothrombosis via oxidant-mediated release of pro-thrombotic factors and activation of platelets?. Front Physiol.

[63] Nemeth E, Rivera S, Gabayan V, Keller C, Taudorf S, Pedersen BK (2004). IL-6 mediates hypoferremia of inflammation by inducing the synthesis of the iron regulatory hormone hepcidin. J Clin Invest.

[64] Rivera S, Nemeth E, Gabayan V, López MA, Farshidi D, Ganz T (2005). Synthetic hepcidin causes rapid dose-dependent hypoferremia and is concentrated in ferroportin-containing organs. Blood.

[65] Bouariu A, Panaitescu AM, Nicolaides KH (2022). First trimester prediction of adverse pregnancy outcomes - Identifying pregnancies at risk from as early as 11-13 weeks. Med Kaunas Lith.

[66] Khambalia AZ, Collins CE, Roberts CL, Morris JM, Powell KL, Tasevski V (2015). High maternal serum ferritin in early pregnancy and risk of spontaneous preterm birth. Br J Nutr.

[67] Jarmund AH, Giske0degard GF, Ryssdal M, Steinkjer B, Stokkeland LMT, Madssen TS (2021). Cytokine patterns in maternal serum from first trimester to term and beyond. Front Immunol.

[68] Farina L, Winkelman C (2005). A review of the role of proinflammatory cytokines in labor and non- infectious preterm labor. Biol Res Nurs.

[69] Preacher KJ, Rucker DD, MacCallum RC, Nicewander WA (2005). Use of the extreme groups approach: A critical reexamination and new recommendations. Psychol Methods.

[70] Thaxton JE, Sharma S (2010). Interleukin-10: A multi-faceted agent of pregnancy. Am J Reprod Immunol.

